# Improving safe street-crossing behaviors among primary school students: a randomized controlled trial

**DOI:** 10.15171/hpp.2018.44

**Published:** 2018-10-27

**Authors:** Hamide Zare, Shamsaddin Niknami, Alireza Heidarnia, Mohamad Hossein Fallah

**Affiliations:** ^1^Department of Health Education,Tarbiat Modares University, Tehran, Iran; ^2^Department of Psychology, Islamic Azad University, Yazd, Iran

**Keywords:** Active learning, ASSURE Model, Children, Pedestrian, Traffic behavior

## Abstract

**Background:** Child pedestrian injury is a global public health concern. Our aim in the present study was to investigate the effects of active learning-based educational intervention on street-crossing behavior among male primary school students in Mehriz county, Iran.

**Methods:** In this randomized controlled trial, 90 first grade elementary school students(experiment = 50, control group = 48) participated. The "ASSURE Model" was applied to design the template of this active learning-based educational program, which was implemented for the experiment group in eight sessions. Behaviors of the students about ‘’looking for vehicles on the street’’, "being cautious of dangers’’ and ‘’crossing from safe places’’ were observed and documented in actual traffic environments before, 1 week, and 6 months after intervention.

**Results:** One week and 6 months after the intervention, the experiment group’s performance in all 3 behaviors were significantly improved (P < 0.001). Distraction-adjusted differences in the mean scores of behaviors between the experiment (Mean = 2.62) and control (Mean = 3.19)groups before and 6 months after intervention (Mean in experiment groups = 6.3, Mean in control group = 4.24) were also statistically significant (P < 0.001).

**Conclusion:** Our educational intervention was found to be helpful in promoting the street crossing behaviors of primary school-aged children. School healthcare professionals may apply active learning education as the core category of their interventional programs to promote street-crossing behaviors among primary school students.

## Introduction

Pedestrian injury is a major cause of child morbidity and mortality throughout the world.^1^ Despite low precision in the global data, car accidents kill over than 30 000 child pedestrians, annually.^2^ According to the Iranian Legal Medicine Organization (LMO) report, from 2006 to 2009, more than 8500 children aged 0–10 years died from pedestrian injuries.^3^

One of the most important reasons for the high rates of child pedestrian injuries is their inadequate cognitive skills.^4^ Despite the fact that cognitive skills in under 9-year-old children are not high enough to enable them for crossing the street, almost all children should perform this behavior on their own when they start going to school.^5,6^ In order to make the street crossing of children safe, several environmental changes have been suggested including designing safe crosswalks on the streets close to schools, traffic calming at school closing times and preventive pedestrian strategies like replacing street crosswalks with pedestrian bridges/tunnels. Among educational interventions conducted to promote the safety of street-crossing behavior among children, the practical training programs for small groups in actual street environments are the most successful programs.^7,8^ Implementing such interventions, however, are reported to be with some limitations (such as high costs), which may be due to the need for multiple educators.^9^ Besides, children education and empowerment for safe street crossing is suggested as an effective strategy to promote this behavior among school children.^10^ In recent years, numerous studies have been conducted utilizing a variety of teaching/learning methods to enhance street-crossing skills among children.^7,11,12^

In Iran, there is a scarcity in the studies on traffic safety education to fewer than 10 years old children. Tabibi in an interventional study with pre- and post-test design and applying a table top model investigated the contributing factors of the Iranian children’s perception of danger on the road, and suggested to increase the risk perception of road crossing among 5-7 years old children.^13^ Almost all the studies conducted in this field evaluated the children applying virtual tools and in virtual environments, which may be due to the potential dangers of real traffic environments.^14^

In order to develop children’s safe street-crossing programs, several teaching/learning theories concerning children’s skills training may be applied. In the present study, constructivist learning theory with a focus on active learning was considered as the theoretical framework to develop the program. Piaget believes that a child’s cognitive growth is the result of interactions between his/her cognitive actions and the surroundings.^15^ This interactional attitude is called “Constructivism”. Based on this theory, a child as an active learner is in complete interaction with the environment to form his complex mental structures, and can therefore resolve his problems on his own.^16^

Active learning is a type of education applied to instruct children in real situations like playing times.^17^ In such real situations, a child may understand the effects of his/her mistakes, and may therefore build his/her mental model based on what he/she has learned. In such learning process, the child performs actively the behavior(s) of interest and the instructor is only a facilitator.^18^ Keeping in mind the Edgar Dale’s Pyramid of Learning, the instructor may observe a considerable improvement in learning and memorizing data by the child during active learning procedure.^19^ Being active during learning may result in high motivation among learners to achieve the learning objectives.^20^ Freeman et al showed that the application of active learning strategy in education improved the results of exams among science, technology, engineering, and mathematics students.^17^ Active learning in our study means to provide children with the conditions that they learn in a game environment. In such an environment, the children observe and percept the impacts of their mistakes and, thus, build and develop their own mental model based on the new learning situations.

In order to design the template of the active learning-based educational program the “ASSURE Model” was applied. Developed by Heinich, Molenda, and Russell,^21^ it is an instructional model that is useful in planning lesson programs. The focus of this model is on the methods of guiding, learning and easing the educational process. The ASSURE Model contains 6 steps according to the constituting letters that form the acronym: Analyze Learner Characteristics, State Objectives, Select Media and Materials, Utilize Media and Materials, Require Learner Participation, and Revise Evaluation.

Checking the experiences of the most successful countries in children’s traffic safety, Christie et al, indicated that all these countries are provided with a comprehensive traffic safety education program implemented in the schools and kindergartens according to the scientific theories of children’s learning.^22^ In Iran, despite the high rates of traffic accident, such a comprehensive programs still are not developed to be implemented at the schools. Pilot studies on such educational programs may be helpful in primary development of traffic safety promotion programs throughout the country. Our aim in the present study was to investigate the effects of active learning-based educational intervention on street-crossing behavior among male primary school students in Mehriz county, Iran.

## Material and Methods

### Study design

This randomized controlled trial (RCT) was conducted from September 2016 to March 2017 in Mehriz a county that is 30 km away from Yazd city, Iran. The study was registered in Iranian Clinical Trials Registry (identifier: IRCT2017031333061N1). The male first-grade elementary-school students with an informed consent form signed by their parents were included in the study. The students who did not attend at least one eighth of the educational program were excluded from the study. Considering the higher rates of traffic accidents among male students in the county, compared to their female counterparts, we decided to focus our trial on the male students, only.

### Sampling

Among all 720 first grade elementary school students in Mehriz, 90 students were invited to participate in the trial. Two out of 10 elementary schools with 4 classes were randomly selected and then were randomly assigned into the experiment and control groups. We considered the mean (standard deviation) of safety traffic behaviors reported by Fyhri et al,^7^ 95% CI, and power of 90% to estimate sample size. The response rates of *P* = 0.3 for the control and *P* = 0.6 for the experiment groups were assumed. The statistical superiority design formula (140* (1+1/720* (1.96^2*0.3*0.7/x^2-1)) - (1.96^2 *0.3* 0.7/x^2) = 0x =0.0681791 (for x! =0)^23^ led us to 41 sample size per group. Considering a potential attrition rate of 20% in each group and the number of students in each class in the schools, two first-grade elementary classes from each primary school (experiment = 50, control group = 48) were selected. The mean of age among all participants was 7.5 years (the age range was 7-8 years).

**Figure 1:**
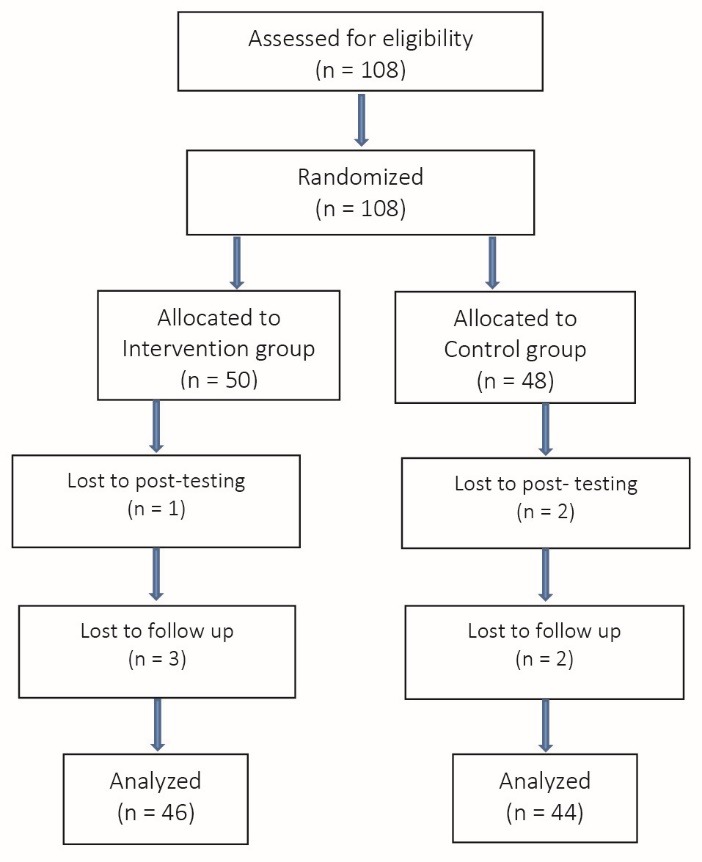
Flowchart of study participants.

The parents of all participants were invited to attend in an introduction session; they signed informed consent forms regarding their child’s participation in the study. During the implementation of study, eight students were excluded, due to their poor participation in the training sessions. Ultimately, data on the behaviors of 46 students in experiment group and 46 students in the control group were analyzed (Figure 1).

### Instruments 

The behavioral test checklist (BTC) was prepared after a review on the previous research. The behavioral assessment questionnaire developed by Zeedyk et al was considered as the framework of the scale.^14^ An expert panel including scholars in the fields of health education and behavior, psychology and police officers reviewed the scale and approved it. The BTS scale included 3 items, in which the students’ behaviors were scored from one to ten. The total score for this scale was from 3-30, in which the higher scores in the scale indicated the more correct behaviors performed by the children. The items were as follow: ‘’looking for vehicles on the street’’, “Being cautious of danger” and ‘’crossing from safe places’’.

### Intervention program 

In this study, we applied the active learning principles^24^ to develop the active learning-based educational program.

### The educational purpose of the program

The educational purpose of the program was to teach the concepts of pedestrians, streets and sidewalks, laws and correct ways of safe street-walking, traffic signs, choosing the safe path, and finally, showing how to pass the street in practice. Different sorts of pictured stories, dolls, toys, roll plays and models were used in accordance to the age and interests of the children.

### The educational package

The educational package of this study was designed to be presented in 8 sessions. The package was based on active learning in actual situations to design the learning situations and to cooperate with the teachers, the interested parents and police officers. The training process was indirectly conducted and the students were active parts of the educational program. Each class contained 25 students. The instructor presented the pictured stories with active participation of the students. The main characters in the stories were mainly the children with the same age and gender. The students roll played instead of the characters and chose on how to behave and react in different traffic situations. As the final stage of the program, the students were participated in a match, and the winners received gifts. For the experiment group, the education was held in eight 45-minute sessions, 2 of which were held in actual environments. The educational program was organized in such a way that the opportunity of participation in the learning situations was equally provided for all students.

### Evaluation of the program

The behaviors of were observed and documented in actual traffic environment and in two 2-way streets near the school, at 3 stages (before, 1 week, and 6 months after implementing the educational program). In order to assess the street-crossing behaviors of the students at the presence of a distracting factor, they were given a pinwheel, as a gift, at the beginning of the second street. Then, their street-crossing behaviors when playing with the toy were investigated.

At the actual situations of the training on the streets, a police officer was present to control the vehicles’ speed and to provide safety for the students. In order to promote the accuracy in scoring the behaviors of the students the whole activates of the students were video-recorded, and the videos were reviewed several times during scoring the behaviors.

### Statistical analyses

Using behavioral assessment questionnaire, data were recorded as 0 (Incomplete behavior) or 1 (Complete behavior) for each items. Then with sum of different items, 3 children behaviors were measured: 1. Looking for vehicles on the street (items 2-5, 8), which range from 1-5, 2. Being cautious of danger (items 1, 7, 10) range from 1-3 and 3. Crossing from safe places (items 6,9), range from 1-2.

Data were entered into and analyzed using IBM SPSS statistics software, version 20 for windows. The outcome variable in our study was the student’s behavior in safe street-crossing. A repeated measurement ANOVA was conducted to assess the effect of educating intervention across different stages (time). As the follow up analysis, post hoc test using Bonferroni adjustment for time and ANCOVA tests for group were used to compare the mean scores of the behaviors in the intervention and control groups before and after the intervention. The differences in the categorical demographic variables between the experiment and control groups were tested using chi-square tests. Paired *t* test was applied to illustrate the variations in the scores on the behaviors before and after intervention. The level of significance was set to be 0.05, a priori.

## Results

No significant difference was found in the demographic variables between the participants of experiment and control groups (Table 1). Before intervention, the behaviors of students, especially in the two cases of ‘’looking for vehicles on the street’’ and ‘’crossing from safe places’’, were weak (Table 2). A repeated measurement ANOVA (with the score on the 3 behaviors as the multiple dependent variable or measurement with one factor group and a within subject factor Time was conducted. The result of repeated measurement ANOVA indicates that the main effect of time [Wilks’ lambda = 0.015, *P* = 0.000] and group [Wilks’ lambda = 0.181, *P*=0.000] are significant. The interaction effect of Time * Group [Wilks’ lambda = 0.024, *P* = 0.000] is also significant. There is no violation from the hypothesis of normality and all dependent variable were correlated (*P* < 0.05). Mauchly’s test indicates that sphericity of covariance matrix is assumed (*P* = 0.13). 

Concerning the significant effect of time and group, post hoc test using Bonferroni adjustment for time and ANCOVA tests are provided in Table 3. The results show that no significant difference was found in the mean scores of the behaviors between the experiment and control groups (*P* > 0.05). One week and 6 months after the intervention, however, the intervention group’s performance in all 3 behaviors of interest were significantly improved (*P* > 0.001). In the control group, the mean scores for the behaviors showed some weak to moderate significant changes. The effects of different stages of the intervention by time on all 3 behaviors were significant. In other words, the performance of the students were found with a linear increasing manner by time (*P* < 0.001).

Table 3 shows the distraction-adjusted differences in the mean scores of behaviors between the experiment and control groups before and 2 stages after intervention. Although the presence of a distracting agent (playing with a pinwheel) reduced the scores of students in the behaviors, the effects of educational intervention were still statistically significant on the behaviors of interest.

## Discussion

Our aim in this study was to determine the effects of active learning-based educational intervention on street-crossing behaviors of the first grade elementary school students. We found the 7-year-old students with extremely unsafe street-crossing behaviors, which reflect lacks in pre-school educations for primary school children in the county. This finding was consistent with those reported by Zeedyk et al, in England^25^ and Tabibi et al, in Iran.^4,13^ Nevertheless, the street-crossing ability of 7-year-old students in our study was similar to that of 5-year-old children in the study conducted in England^14^ which may be due to the differences in education methods, and/or observational learning from unsafe behaviors of adults, and the low safety of streets’ environment around the schools in Iran.

Findings of the present study represented that one week after implementing the educational intervention, all 3 behaviors in the experiment group were significantly improved, compared to the control group, and the improvement almost persisted after 6 months. This result was in line with the finding of Zeedyk et al,^14^ Albert and Dolgin,^12^ and Fyhri et al.^7^ We also found that the effects of active learning-based education were still significant on the behaviors of interest, even at the presence of a distraction agent. This findings indicate that active learning-based education may relieve the influence of distraction factors on children’s attention when crossing streets, as announced in previous studies.^14,26^ There are also several previous studies that suggest practical training, individually or in small groups, with the greatest success in promoting children’s safe behaviors.^12,27,28^

Although the theoretical instructions or using video or computer games in classrooms may promote the knowledge of school-age children, without practical learning in actual situations there would be a lack of success in improving the traffic-related behavioral skills of children under the age of 9.^27,29^ These previously stated claims were intensely confirmed in the present study, as active learning through indirect learning as well as child involvement in the learning process led the students to progress in street-crossing behaviors.

Evaluating the behaviors of students 6 months after the intervention indicated no significant decline on the student’s performance in all 3 behaviors. In contrast, the most of previous studies reported significant decrease in the impacts of educational programs on traffic safety behaviors two months after the intervention.^30,31^ Considering the impacts of observation and then imitation from others in the learning process among children, it may be expected to find decrease in the level of safe street-crossing behaviors among the students. The school-age children are daily witnessing some degrees of inappropriate traffic-related behaviors among a majority of adult traffic users. The students, therefore, may be influenced by the unsafe behaviors of significant others through observational learning, and not to perform the street-crossing behaviors in a safe manner. However, the safety behaviors of interest in our study were not remarkably decreased among the students after 6 months which may indicate the significant effects of active learning-based education on instant behavior change among children.

In the present study, we also found a relative improvement in the behaviors of students in the control group. There may be 2 reasons for this finding; school routine education and age. The students in the control group may be instructed on the behaviors during their school routine education. The growing process of children may also be another reason, considering that Schwebel et al^29^ and Barton et al^32^ reported age as one of the most important determinants for traffic safety behaviors among children.

Considering the significant influence of street environment on the behaviors of children, the two experimental groups were selected from a school, which may prone the findings to contamination bias. Due to this limitation, the results may not be generalizable to other settings. Two months after implementing the intervention, a post-test was considered; however, due to the timing of school exams and the lack of cooperation from the school in the control group, the results of this post-test were excluded from the findings.

In order to design effective educational programs, school healthcare professionals should better understand the determinants of safe street-crossing behaviors among the primary schools students and develop stage-specific interventions, within which active learning education is the core category of the program.

## Conclusion

The application of ASSURE Model as the framework of educational intervention was found to be helpful in analyzing the students’ characteristics, utilizing the media and materials, and involving the students in the learning processes. The model was also useful in addressing the effects of active-learning-based educational intervention on street-crossing behaviors of primary school-aged children. Such an application of the instructional models, as a framework for planning active learning-based educational programs among school-age children, may be useful in improving the students’ street-crossing behaviors in actual learning settings.

## Ethical approval

This study was approved by Research Ethics Committee in Faculty of Medical Sciences, Tarbiat Modares University, Tehran, Iran (IR.TMU.REC.1394.49). After explaining the procedure of the study, written informed consent was obtained from all parents of the participants.

## Informed Consent

Informed consent was obtained from the parents of all participants included in the study.

## Competing interests

The authors declare no conflicts of interest.

## Authors’ contributions

Study conception was conducted by SN, HZ and AH. HZ collected the data. MHF performed analyses. HZ drafted the manuscript. All authors have reviewed and provided intellectual feedback on the manuscript. All authors have read and approved the final version of the manuscript and agree with the order of presentation of the authors.

## Acknowledgements

We thank all those helped us to conduct the study, including Mehriz traffic police, and the school principals, teachers and authorities. 

**Table 1:** Demographic characteristics of the students participated in the experiment and control groups

**Variables**	**Range**	**Experiment**	**Control**	***P*** ** value**
Age, mean (SD)		7.59 (0.49)	7.57 (0.5)	0.859
Family size, %	3	54.7	45.3	0.867
	4	51.5	48.5	
	5	41.8	58.2	
	6	50	50	
Mother’s job,%	Employee	50	50	0.632
	Housewife	59.2	40.8	
	Self-employed	33	67	
Mother’s education,%	<High school	50	50	0.144
	High school	37	63	
	Academic degree	65.3	34.7	

**Table 2:** Differences in the mean scores of the behaviors between the experiment and control groups before and 2 stages after intervention

**Variables**	**Time**	**Experiment group** **(n = 46)**		**Control group** **(n = 44)**		***P*** **value** ^b^	**Maximum**
		**Mean scores**	**SD**	**Mean scores**	**SD**		
Looking for vehicles on the street	1- Before intervention	0.83	0.28	0.75	0.26	0.061	5
	2- One week after intervention	1.94	1.47	1.82	0.27	< 0.001	
	3- 6 months after intervention	1.55	0.65	1.02	0.8	< 0.001	
	Post hoc test’s for time	(1-2,<0.001) , (1-3,<0.001) , (2-3,<0.007)^a^ ,							
Being cautious of dangers	1- Before intervention	1.61	0.8	1.63	0.78	0.560	3
	2- One week after intervention	2.36	0.61	1.64	0.71	< 0.001	
	3- 6 months after intervention	2.21	0.61	1.89	0.59	< 0.001	
	Post hoc test’s for time	(1-2,<0.001) , (1-3,<0.001) , (2-3, 0.955)^a^							
Crossing from safe places	Before intervention	0.18	0.12	0.81	0.39	0.031	2
	One week after intervention	2	0.36	0.82	0.38	< 0.001	
	6 months after intervention	1.74	0.44	0.92	0.26	< 0.001	
	Post hoc test’s for time	(1-2,<0.001) , (1-3,<0.001) , (2-3, 0.29)^a^							

^a^ Post hoc test’s, Bonferroni adjustment for multiple comparisons. ^b^ F test for ANCOVA.

**Table 3:** Distraction-adjusted differences in the mean scores of behaviors between the experiment and control groups before and 2 stages after intervention

**Variables**	**Time**	**Experiment**		**Control**		***P*** ** value**	**Maximum**
		**Mean scores**	**SD**	**Mean scores**	**SD**		
Looking for vehicles on the street	Before intervention	0.11	0.31	0.15	0.42	0.081	5
	One week after intervention	1.77	1.4	0.19	0.25	< 0.0001	
	6 months after intervention	1.41	0.85	0.85	0.41	< 0.0001	
	Post hoc test’s for time	(1-2,<0.0001) , (1-3,<0.001) , (2-3,<0.25)^a^ ,							
Being cautious of dangers	Before intervention	0.41	0.67	0.79	0.85	0.23	3
	One week after intervention	2.04	0.85	0.86	0.89	< 0.0001	
	6 months after intervention	1.86	0.61	1.01	0.68	< 0.0001	
	Post hoc test’s for time	(1-2,<0.001) , (1-3,<0.001) , (2-3,<0.001)^a^ ,							
Crossing from safe places	Before intervention	0	0	0	0	0.206	2
	One week after intervention	1.75	0.26	0.4	0.12	< 0.0001	
	6 months after intervention	1.4	0.41	0.71	0.68	< 0.002	
	Post hoc test’s for time	(1-2,<0.001) , (1-3,<0.001) , (2-3,<0.96)^a^							

^a^ Post hoc test’s, Bonferroni adjustment for multiple comparisons. ^b^ F test for ANCOVA.
